# Antifibrinolytic Agents in Traumatic Haemorrhage

**DOI:** 10.1371/journal.pmed.0020064

**Published:** 2005-03-29

**Authors:** Tim Coats, Beverly Hunt, Ian Roberts, Haleema Shakur

## Abstract

Among trauma patients who survive to reach hospital, exsanguination is a common cause of death. Could anti fibrinolytics reduce the death rate? Only a large randomized controlled trial can answer the question

## Teaser

A large-scale randomised controlled trial is needed

## Introduction

This article is an invitation to doctors around the world to participate in the CRASH-2 trial (Clinical Randomisation of an Antifibrinolytic in Significant Haemorrhage), a large, multi-centre, randomised controlled trial of a simple and widely practicable treatment for traumatic hemorrhage. The rationale for the trial and contact details for those who would like to take part are given below.

Evidence from randomised controlled trials is essential for improving health care. In the case of widely practicable treatments for common health problems, even modest treatment effects can result in substantial health gains. However, to detect such modest effects requires large multi-centre randomised trials involving hundreds of collaborating health professionals internationally. Many health professionals would be pleased to collaborate in such trials if they knew that they were underway, but at present there is no easy way to bring these trials to their attention.

Three years ago, in the context of the CRASH-1 trial (Corticosteroid Randomisation After Significant Head Injury), the trial investigators sent a message to the electronic mailing list of the World Association of Medical Editors, asking them to consider publishing an editorial about the CRASH-1 trial that invited doctors around the world to participate. In response to this request, many medical journals around the world published the CRASH-1 trial editorial in various different languages, and as a result, many more doctors joined the CRASH-1 trial. The trial was completed in May 2004 and involved around 400 hospitals in almost 50 countries, and because of its size provided a reliable answer to an important question (see www.crash.lshtm.ac.uk).

This current article is being published as the result of a similar such request to medical editors in the context of the CRASH-2 trial.

## A Possible Role for Antifibrinolytics

For people at ages five to 45 years, trauma is second only to HIV/AIDS as a cause of death. Each year, worldwide, over three million people die as a result of trauma, many after reaching hospital [1]. Among trauma patients who do survive to reach hospital, exsanguination is a common cause of death, accounting for nearly half of in-hospital trauma deaths [2]. Central nervous system injury and multi-organ failure account for most of the remainder, both of which can be exacerbated by severe bleeding [3].

The haemostatic system helps to maintain the integrity of the circulatory system after severe vascular injury, whether traumatic or surgical in origin [4]. Major surgery and trauma trigger similar haemostatic responses, and any consequent massive blood loss presents an extreme challenge to the coagulation system. Part of the response to surgery and trauma, in any patient, is stimulation of clot breakdown (fibrinolysis) which may become pathological (hyper-fibrinolysis) in some [4]. Antifibrinolytic agents have been shown to reduce blood loss in patients with both normal and exaggerated fibrinolytic responses to surgery, and do so without apparently increasing the risk of post-operative complications; most notably there is no increased risk of venous thromboembolism [5].

Systemic antifibrinolytic agents are widely used in major surgery to prevent fibrinolysis and thus reduce surgical blood loss. A recent systematic review [6] of randomised controlled trials of antifibrinolytic agents (mainly aprotinin or tranexamic acid) in elective surgical patients identified 89 trials including 8,580 randomised patients (74 trials in cardiac, eight in orthopaedic, four in liver, and three in vascular surgery). The results showed that these treatments reduced the numbers needing transfusion by one third, reduced the volume needed per transfusion by one unit, and halved the need for further surgery to control bleeding. These differences were all highly statistically significant. There was also a statistically non-significant reduction in the risk of death (relative risk = 0.85: 95% confidence interval, 0.63–1.14) in the antifibrinolytic-treated group.

## Why a Large Trial Is Needed

Because the haemostatic abnormalities that occur after injury are similar to those after surgery, it is possible that antifibrinolytic agents might also reduce blood loss, the need for transfusion and mortality following trauma. However, to date there has been only one small randomised controlled trial (70 randomised patients, drug versus placebo: zero versus three deaths) of the effect of antifibrinolytic agents in major trauma [7]. As a result, there is insufficient evidence to either support or refute a clinically important treatment effect. Systemic antifibrinolytic agents have been used in the management of eye injuries where there is some evidence that they reduce the rate of secondary haemorrhage [8].

A simple and widely practicable treatment that reduces blood loss following trauma might prevent thousands of premature trauma deaths each year, and secondly, could reduce exposure to the risks of blood transfusion. Blood is a scarce and expensive resource, and major concerns remain about the risk of transfusion-transmitted infection. Trauma is common in parts of the world where the safety of blood transfusion is not assured. A recent study in Uganda estimated the population-attributable fraction of HIV acquisition as a result of blood transfusion to be around 2%, although some estimates are much higher [9,10]. Only 43% of the 191 WHO member states test blood for HIV, Hepatitis C, and Hepatitis B viruses. Every year, unsafe transfusion and injection practices are estimated to account for 8–16 million Hepatitis B infections, 2.3–4.7 million Hepatitis C infections, and 80,000–160,000 HIV infections [11]. A large randomised trial is therefore needed of the use of a simple, inexpensive, widely practicable antifibrinolytic treatment such as tranexamic acid (aprotinin is considerably more expensive and is a bovine product with consequent risk of allergic reaction and hypothetically transmission of disease), in a wide range of trauma patients, who when they reach hospital are thought to be at risk of major haemorrhage that could significantly affect their chances of survival.

## A Call to Health Professionals

The CRASH-2 trial will be a large, international, placebo-controlled trial of the effects of the early administration of the antifibrinolytic agent tranexamic acid on death, vascular events and transfusion requirements (http://www.crash2.lshtm.ac.uk). The trial aims to recruit some 20,000 patients with trauma and will be one of the largest trauma trials ever conducted. However, it will only be possible to conduct such a trial if hundreds of health care professionals worldwide work together to recruit patients to the trial in order to make it a success. If you are interested in recruiting patients, please contact Ian Roberts at the CRASH-2 trial coordinating centre (Box 1).

**Box 1. Contact Information for the CRASH-2 Trial**

Ian Roberts
CRASH-2 Trial Co-Ordinating Centre
London School of Hygiene and Tropical Medicine
Keppel Street, London WC1E 7HT
Phone: 0207 958 8128
Fax: 0207 299 4663
Web site: www.crash2.lshtm.ac.uk
E-mail: Ian.roberts@lshtm.ac.uk

*A similar version of this article is being published in several medical journals worldwide.*

**Figure:**
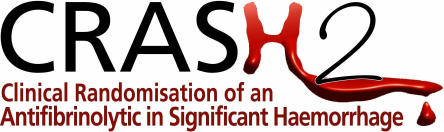


## Abbreviations

CRASH-1: Corticosteroid Randomisation After Significant Head Injury
CRASH-2: Clinical Randomisation of an Antifibrinolytic in Significant Haemorrhage

## References

[pmed-0020064-b1] Murray CJL, Lopez AD (1996). Global health statistics: A compendium of incidence, prevalence and mortality estimates for over 200 conditions.

[pmed-0020064-b2] Sauaia A, Moore FA, Moore E, Moser K, Brennan R (1995). Epidemiology of trauma deaths: A reassessment. J Trauma.

[pmed-0020064-b3] The Brain. Trauma Foundation. The American Association of Neurological Surgeons. The Joint Section on Neurotrauma and Critical Care (2000). Hypotension. J Neurotrauma.

[pmed-0020064-b4] Lawson JH, Murphy MP (2004). Challenges for providing effective hemostasis in surgery and trauma. Semin Hematol.

[pmed-0020064-b5] Porte RJ, Leebeek FW (2002). Pharmacological strategies to decrease transfusion requirements in patients undergoing surgery. Drugs.

[pmed-0020064-b6] Henry DA, Moxey AJ, Carless PA, O'Connell D, McClelland B (2004). Anti-fibrinolytic use for minimising perioperative allogeneic blood transfusion. Cochrane Database Syst Rev.

[pmed-0020064-b7] Coats T, Roberts I, Shakur H (2004). Antifibrinolytic drugs for acute traumatic injury. Cochrane Database Syst Rev.

[pmed-0020064-b8] Aylward GW, Dunlop IS, Little BC (1994). Meta-analysis of systemic antifibrinolytics in traumatic hyphema. Eye.

[pmed-0020064-b9] Kiwanuka N, Gray RH, Serwadda D (2004). The incidence of HIV-1 associated with injections and transfusions in a prospective cohort, Raki, Uganda. AIDS.

[pmed-0020064-b10] Heymann SJ, Brewer TF (1992). The problem of transfusion associated acquired immunodeficiency syndrome in Africa: a quantitative approach. Am J Infect Control.

[pmed-0020064-b11] Goodnough LT, Shander A, Brecher ME (2003). Transfusion medicine: Looking to the future. Lancet.

